# Plant cell adhesion and growth on artificial fibrous scaffolds as an in vitro model for plant development

**DOI:** 10.1126/sciadv.abj1469

**Published:** 2021-10-20

**Authors:** Ryan Calcutt, Richard Vincent, Derrick Dean, Treena Livingston Arinzeh, Ram Dixit

**Affiliations:** 1 Department of Biology and Center for Engineering Mechanobiology, Washington University in St. Louis, St. Louis, MO 63130, USA.; 2 Department of Biomedical Engineering and Center for Engineering Mechanobiology, New Jersey Institute of Technology, Newark, NJ 07102, USA.; 3 Biomedical Engineering Program and Center for Engineering Mechanobiology, Alabama State University, Montgomery, AL 36014, USA.

## Abstract

Mechanistic studies of plant development would benefit from an in vitro model that mimics the endogenous physical interactions between cells and their microenvironment. Here, we present artificial scaffolds to which both solid- and liquid-cultured tobacco BY-2 cells adhere without perturbing cell morphology, division, and cortical microtubule organization. Scaffolds consisting of polyvinylidene tri-fluoroethylene (PVDF-TrFE) were prepared to mimic the cell wall’s fibrillar structure and its relative hydrophobicity and piezoelectric property. We found that cells adhered best to scaffolds consisting of nanosized aligned fibers. In addition, poling of PVDF-TrFE, which orients the fiber dipoles and renders the scaffold more piezoelectric, increased cell adhesion. Enzymatic treatments revealed that the plant cell wall polysaccharide, pectin, is largely responsible for cell adhesion to scaffolds, analogous to pectin-mediated cell adhesion in plant tissues. Together, this work establishes the first plant biomimetic scaffolds that will enable studies of how cell-cell and cell-matrix interactions affect plant developmental pathways.

## Teaser

Artificial fibrous matrices that mimic the plant cell wall enable the adhesion and culture of plant cells in vitro.

## INTRODUCTION

Development of multicellular organisms involves generating different cell types at specific locations within tissues and organs. In both plants and animals, cell lineage and positional information play a critical role in cell-fate control and tissue patterning (*1*–*3*). Coordination between cells is achieved through cell-cell communication that is mediated either by direct interaction of cells or by biochemical and mechanical signals (*4*, *5*). Cellular responses to developmental signals can, in turn, feedback to affect the growth and behavior of neighboring cells (*6*–*10*). Thus, multicellular context and recursive feedback between cells and their environment are major components of plant and animal development.

While whole-organism studies have laid the foundation to understanding the development of multicellular organisms, dissecting the complex interplay between genetic, biochemical, and mechanical inputs that operate at multiple length and time scales is challenging using this approach. In mammalian systems, the fabrication of organoids has been pivotal in overcoming this barrier. Organoids recapitulate the anatomical and physiological complexity of tissues and organs under defined conditions, thus providing experimentally tractable in vitro models of human development (*11*, *12*). The creation of organoids relies on appropriate substrates for the attachment, proliferation, and differentiation of cells into desired three-dimensional tissue configurations (*13*–*15*). Substrates play a critical and active role in organoid formation by providing the necessary biochemical and mechanical cues that guide cell-cell and cell-matrix interactions. Ideal substrates mimic the physical and chemical properties of endogenous tissues to form durable and physiologically accurate organoids. In addition, altering the material properties of substrates provides nuanced control of the physical and biochemical microenvironment to probe mechanisms underlying the development and physiology of organoids (*16*, *17*).

The plant body consists largely of diffusively growing cells that are attached to their neighbors by their cell walls. The rigid cell wall provides structural support as well as mechanical and biochemical cues that guide cell morphogenesis and development (*6*, *9*, *10*, *18*–*20*). The physical coupling of cells by the cell wall also enables direct force transmission to coordinate the growth of adjoining cells in developing tissues and organs (*6*, *20*, *21*). The primary cell wall of growing plant cells is a fibrous composite material consisting of cable-like cellulose microfibrils embedded within a matrix of hemicellulose and pectin polysaccharides. Cellulose microfibrils consist of crystalline chains of β-1,4 glucan and are the major load-bearing elements of plant cell walls (*22*, *23*). Hemicelluloses are branched polysaccharides that associate noncovalently with cellulose microfibrils to form a cross-linked network (*24*). Pectins are complex, polyanionic polymers that contribute to the porosity and extensibility of the cell wall through crosslinking by borate or Ca^2+^ ions (*19*, *25*, *26*, *27*). Pectins are also a major component of the middle lamella that glues the cell walls of adjacent plant cells to provide tissue integrity (*28*). The primary cell wall also contains a small amount of proteins that contribute to wall structure, signaling, and wall remodeling (*29*–*31*).

Accruing evidence has firmly established that plant development is responsive to internal and external mechanical cues (*6*, *9*, *10*, *20*, *21*, *32*). Mechanical forces can initiate biochemical signaling and regulate gene expression. Reciprocally, genetic and biochemical activities can generate or modify mechanical cues. However, we have little understanding of the interplay between these factors and their relative contribution to plant development. To address this critical knowledge gap, we need a simple and controllable in vitro model system akin to mammalian organoids and tissue engineering models. Currently, the most common methods for in vitro culture of plant cells consist of growth on agar surface in the form of an unorganized mass of undifferentiated cells called a callus or as suspension cultures in liquid medium. However, none of the existing in vitro culture techniques mimic the tissue structure and microenvironment experienced by cells in the plant body. In addition, these techniques are not amenable to impose spatially graded mechanical stimuli or create local hormone maxima, which are known to be important regulators of plant development. To overcome these limitations, we need substrates that mimic the endogenous plant extracellular environment on which functional plant tissues can be engineered.

In this study, we report artificial scaffolds that support the adhesion and viable culture of growing plant cells. These scaffolds are made of polyvinylidene tri-fluoroethylene (PVDF-TrFE), which closely mimics the fibrous structure, relative hydrophobicity, and piezoelectric property of plant cell walls. We show that cultured tobacco Bright Yellow-2 (BY-2) cells adhere tightly to these scaffolds without adversely affecting their growth, morphology, and division and that the cell-scaffold adhesion involves pectin.

## RESULTS

### Plant cell adhesion to scaffolds that vary in fiber dimension and orientation

**Fig. 1.**
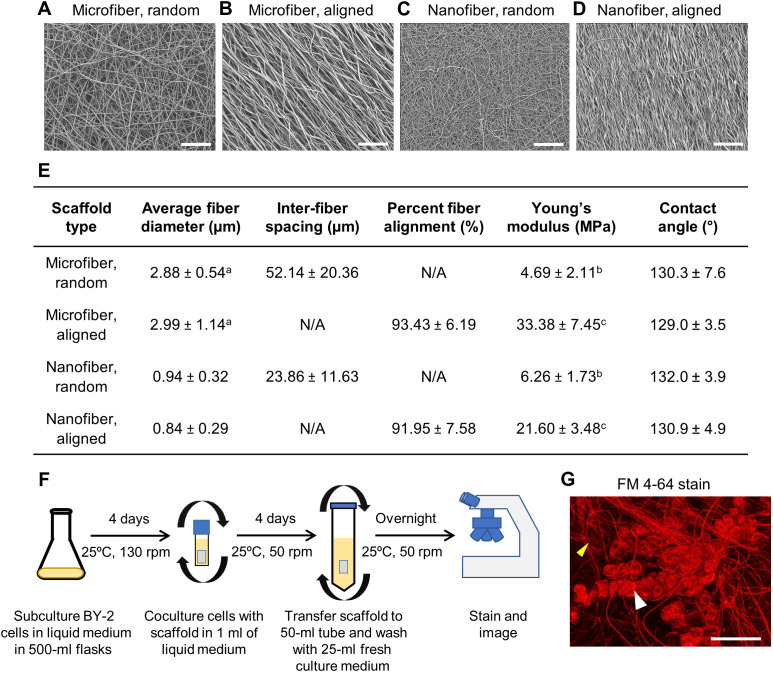
Adhesion of tobacco BY-2 cells to PVDF-TrFE scaffolds. (**A** to **D**) Scanning electron micrographs of PVDF-TrFE scaffolds used in this study. Scale bars, 100 μm. (**E**) Table showing the fiber diameter, inter-fiber spacing, fiber alignment, Young’s modulus, and air-water contact angle of the PVDF-TrFE scaffolds. Values are means ± SD (*n* = 15 per sample group for fiber diameter and spacing measurements; *n* = 20 for fiber alignment measurements; *n* = 10 for the mechanical measurements; *n* = 4 for contact angle measurements). ^a^*P* < 0.05, significantly different from PVDF-TrFE nanofiber group; ^b^*P* < 0.05, significantly different from aligned groups; ^c^*P* < 0.05, significantly different from all other groups. N/A, not applicable. (**F**) Outline of the screening procedure used to determine whether cultured tobacco BY-2 cells adhere to PVDF-TrFE scaffolds. (**G**) Fluorescence micrograph of BY-2 cells attached to a microfiber randomly oriented PVDF-TrFE scaffold stained with FM 4-64. The white arrowhead points to BY-2 cells, while the yellow arrowhead points to scaffold fibers stained by the FM 4-64 dye.

At the ultrastructural level, the plant cell wall consists of fibers that vary in dimension and alignment (*33*, *34*). To examine whether the diameter and spatial organization of scaffold fibers influence the adhesion of plant cells, we fabricated PVDF-TrFE scaffolds consisting of either nano- or micrometer-sized fiber diameters that were either random or aligned in orientation (Fig. 1, A to E). To determine whether plant cells adhered to these scaffolds, we used a suspension culture of tobacco BY-2 cells derived from *Nicotiana tabacum* L. cv. BY-2. Tobacco BY-2 cells are widely used as a plant cell model system because of their fast growth rate, ease of live imaging, and ability to generate stable transgenic lines using *Agrobacterium*-mediated stable transformation (*35*). Tobacco BY-2 cells 4 days after subculture were coincubated with 0.75 cm–by–1 cm pieces of sterilized scaffolds in 1 ml of liquid BY-2 culture medium. Tubes were kept on a rotary shaker for 4 days to allow cell adhesion, followed by a vigorous overnight wash with 25 ml of fresh BY-2 culture medium in a 50-ml tube to remove unattached cells (Fig. 1F). Scaffolds were then stained with the lipophilic styryl dye FM 4-64 to visualize any adhered cells using confocal microscopy (Fig. 1G). The FM 4-64 dye rapidly labels the plasma membrane of cells and subsequently labels endomembrane compartments due to uptake by endocytosis (*36*). We found that BY-2 cells adhered to all four types of PVDF-TrFE scaffolds.

**Fig. 2.**
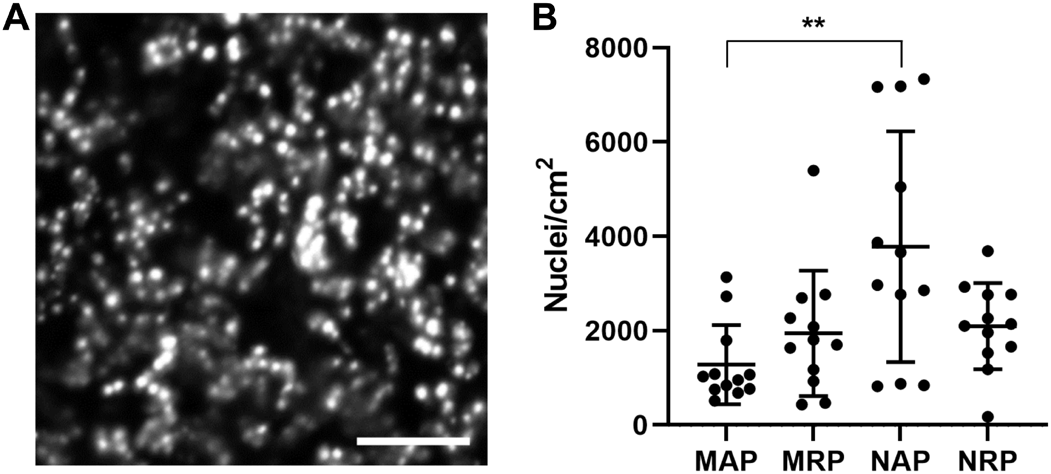
Quantifying the extent of cell adhesion to scaffolds. (**A**) Maximum intensity projection image of tobacco BY-2 cell nuclei stained with DAPI on a microfiber randomly oriented PVDF-TrFE scaffold. Scale bar, 100 μm. (**B**) Quantification of cell nuclei found on microfiber aligned PVDF-TrFE (MAP), microfiber randomly oriented PVDF-TrFE (MRP), nanofiber aligned PVDF-TrFE (NAP), and nanofiber randomly oriented PVDF-TrFE (NRP). Center line and error bars indicate means ± SD from 12 independent biological replicates for each type of scaffold. The means between MAP and NAP scaffolds are statistically different as determined by the Mann-Whitney test with Bonferroni correction for multiple comparisons (*P* = 0.007).

We sought to quantify the cell count on each type of scaffold. While the FM 4-64 staining revealed attached cells, it was challenging to distinguish individual cells using this dye, particularly in dense fields. In addition, this dye stained the fibers of the scaffolds (Fig. 1G), further hampering cell quantification. To overcome these problems, we used DAPI (4′,6-diamidino-2-phenylindole) to stain cell nuclei and developed an ImageJ macro to count the number of discrete nuclei on each scaffold as a proxy for cell abundance (Fig. 2A). This quantification revealed the PVDF-TrFE scaffolds had an average of approximately 2000 cells/cm^2^ attached (Fig. 2B). We found that the nanofiber aligned PVDF-TrFE (NAP) scaffolds had significantly higher mean cell counts than the microfiber aligned PVDF-TrFE scaffolds (MAP) (Fig. 2B). While the NAP scaffolds had higher mean cell counts than the microfiber and nanofiber randomly oriented PVDF-TrFE (MRP and NRP, respectively) scaffolds, these differences were not statistically distinguishable. In addition, although the PVDF-TrFE scaffolds with aligned and random fiber arrangements differ in Young’s moduli (Fig. 1E), cell attachment was not affected. All scaffolds were also relatively hydrophobic as measured by air-water contact angle using a goniometer (Fig. 1E).

### Adhesion to scaffold does not affect cell morphology

**Fig. 3.**
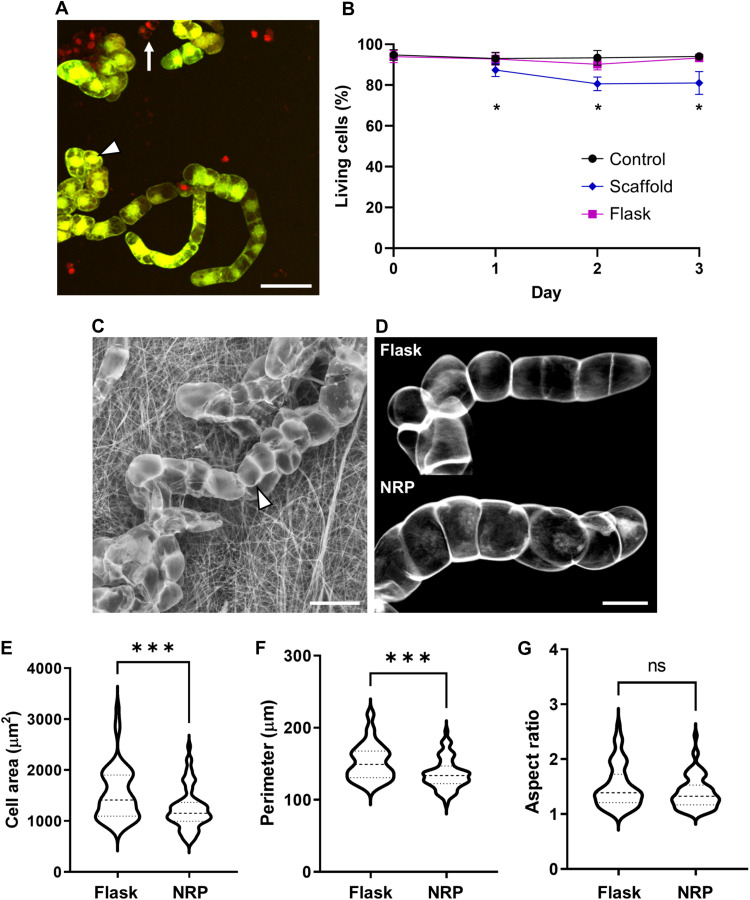
Analysis of cell viability and morphology on scaffolds. (**A**) Micrograph of BY-2 cells stained with fluorescein diacetate (green, arrowhead) and propidium iodide (red, arrow). Fluorescein diacetate stains live cells, while propidium iodide stains dead cells. (**B**) Percentage of viable cells over time. The viability of BY-2 cells attached to NRP scaffolds and unattached in the same flask was determined 1, 2, and 3 days after coincubation of NRP scaffolds with BY-2 cells. Control cells were grown in liquid medium without a scaffold. Values indicate means ± SD (*n* = 3 biological replicates, each with four technical replicates). *P < 0.02 using ANOVA. (**C**) Environmental scanning electron micrograph of BY-2 cells attached to the NRP scaffold. Scale bar, 40 μm. (**D**) Average intensity projection images of BY-2 cells stained with calcofluor that were grown either without a scaffold in liquid culture (flask) or on the NRP scaffold. Scale bar, 20 μm. (**E** to **G**) Violin plots of cell size (E), perimeter (F), and aspect ratio (G) of cells grown in liquid culture (flask, *n* = 53) or on the NRP scaffold (*n* = 68). ***P < 0.001 using ANOVA; ns, not significant.

To determine whether attachment to PVDF-TrFE scaffolds affects cell viability, we compared the percentage of living cells over time between liquid culture and scaffold grown conditions. Cell viability was assessed by staining with a combination of fluorescein diacetate and propidium iodide. Fluorescein diacetate stains the cytoplasm of living cells, whereas propidium iodide labels the DNA of dead cells by passing through damaged cell membranes (Fig. 3A) (*37*). Cells attached to scaffolds showed a small decrease in viability compared to unattached cells coincubated with scaffolds and control cells grown in liquid medium without a scaffold (Fig. 3B). Nonetheless, approximately 80% of cells attached to a scaffold were alive over 3 days.

During our screening process, we observed that BY-2 cells predominantly adhered to the surface of scaffolds and only occasionally were found to be enmeshed within scaffold fibers (Fig. 3C and fig. S1). Cells attached to scaffolds appeared morphologically similar to control cells grown in flasks in the absence of scaffolds. We saw normal-looking BY-2 cell files when stained with the FM 4-64 dye (Fig. 1G) and when imaged using an environmental scanning electron microscope (SEM; Fig. 3C). To quantitatively compare the size and shape of BY-2 cells adhered to scaffolds versus those grown in liquid medium alone, we stained cells with calcofluor white, which binds to cellulose and labels the outline of cells (Fig. 3D). We found that BY-2 cells grown on NRP scaffolds had a slightly smaller area and perimeter compared to cells of the same age grown in flasks (Fig. 3, E and F). However, the length-to-width aspect ratio of cells was not significantly different between the two conditions (Fig. 3G), indicating that cell morphology was not significantly affected by scaffold attachment. This finding is in contrast to a previous study that used scaffolds containing a mix of poly(ethylene terephthalate) (PET) microfibers and polylactide (PLA) nanofibers, which resulted in abnormal plant cell morphology (*38*).

### Characterization of the growth, division, and cortical microtubule dynamics of cells on scaffolds

**Fig. 4.**
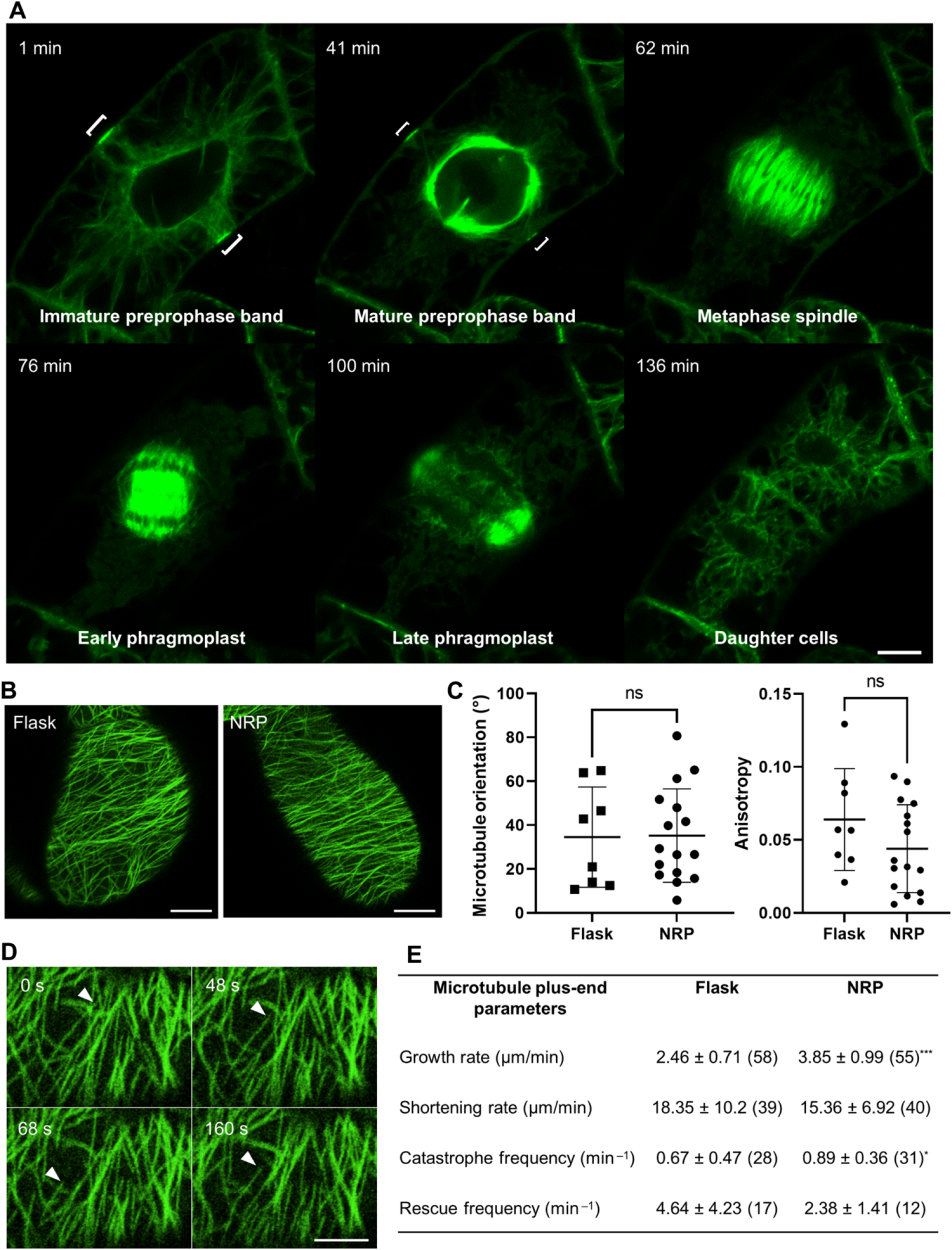
Cell division and microtubule dynamics in cells attached to a scaffold. (**A**) Fluorescence micrograph of microtubules labeled by GFP-TUB6 at different stages of mitosis in a BY-2 cell attached to the NRP scaffold. Scale bar, 10 μm. (**B**) Maximum intensity projections of cortical microtubules in cells grown either without a scaffold in liquid culture (flask) or on the NRP scaffold. Scale bars, 10 μm. (**C**) Cortical microtubule array orientation and anisotropy measured from individual cells grown in liquid culture (flask) or on the NRP scaffold. Center line and error bars indicate means ± SD (flask, *n* = 8 cells; NRP, *n* = 16 cells). (**D**) Example of microtubule polymerization dynamics in a cell grown on the NRP scaffold. Arrowheads label the plus end of the microtubule of interest. Scale bar, 5 μm. (**E**) Dynamics of the plus ends of cortical microtubules. Values are means ± SD (*n* = number of events). ***P < 0.0001 and *P < 0.005 as determined by the Mann-Whitney test.

To be suitable for plant developmental studies, scaffolds must be able to support growth and division of cells. To visualize cell division, we generated a transgenic BY-2 cell line expressing green fluorescent protein (GFP)–labeled β-tubulin 6 (GFP-TUB6) driven by the constitutive *Arabidopsis thaliana ubiquitin-10* promoter. To observe dividing cells before the culture reached stationary phase, we cocultured NRP scaffolds with GFP-TUB6–expressing BY-2 cells 2 days after subculture and imaged cells on scaffolds 4 days later. Under these conditions, we captured 20 cells undergoing mitosis over three independent experiments. Examination of cell division using time-lapse fluorescence microscopy revealed that the major mitotic microtubule structures are normal in cells attached to NRP scaffolds (Fig. 4A). In addition, phragmoplasts accurately inserted at cortical division sites marked by the preprophase band, and we did not observe any abnormal cell division orientations, indicating that cytokinesis was unaffected by adhesion of cells to the scaffold.

Next, we observed cortical microtubules in interphase cells. Both flask-grown and NRP scaffold–attached cells showed various cortical microtubule orientations that correlated with cell shape (Fig. 4B). In non-elongating cells in stationary phase and in ovoid cells, cortical microtubules had mixed orientations or were oriented parallel to the long axis of the cell. By contrast, cortical microtubules were oriented predominantly perpendicular to the long axis of elongated cells. These findings are consistent with the known role of cortical microtubules in defining the axis of cell expansion by directing the deposition of cellulose microfibrils (*39*). Using the FibrilTool ImageJ plugin (*40*), we found that the overall microtubule array orientation and degree of anisotropy were similar between flask-grown cells and cells attached to the NRP scaffold (Fig. 4C). We tracked the polymerization dynamics of the plus-ends of cortical microtubules (Fig. 4D) and found that cortical microtubules in NRP scaffold–attached cells showed an increased growth rate and catastrophe frequency compared to cells grown in liquid alone, while the shortening rate and rescue frequency were not significantly different (Fig. 4E).

**Fig. 5.**
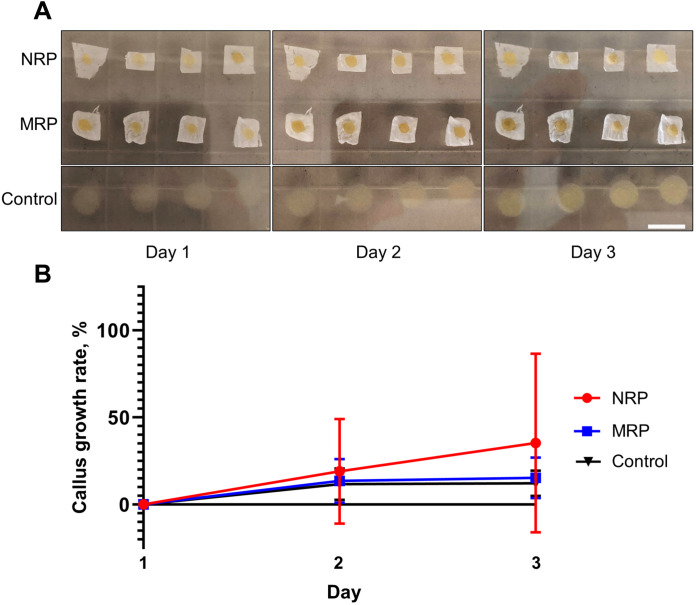
Growth of BY-2 callus cells on the surface of scaffolds. (**A**) Images of BY-2 cells inoculated either on the surface of the indicated scaffolds or directly on the agar medium (control). Scale bar, 10 mm. Photo credit: Ryan Calcutt, Washington University in St. Louis. (**B**) Graph of callus growth rate on scaffolds and on agar (control). Values are means ± SD (*n* = 15 for NRP, *n* = 16 for MRP and control). Comparison of the values using a Kruskal-Wallis test with a Dunn’s test for multiple comparison showed that callus growth on the scaffolds is indistinguishable from the control.

To determine whether these scaffolds can be used for solid medium plant tissue culture, we inoculated BY-2 cells on the surface of microfiber randomly oriented and nanofiber randomly oriented scaffolds placed on solidified culture medium. As a control, BY-2 cells were inoculated directly on the surface of the agar medium. BY-2 cells grew as callus tissue both on the surface of the scaffolds and on agar (Fig. 5A). By measuring the area of individual calli over time, we found that the callus growth rate was not significantly different between the scaffolds and agar (Fig. 5B).

### Dipole orientation in piezoelectric PVDF-TrFE scaffolds contribute to plant cell attachment

**Fig. 6.**
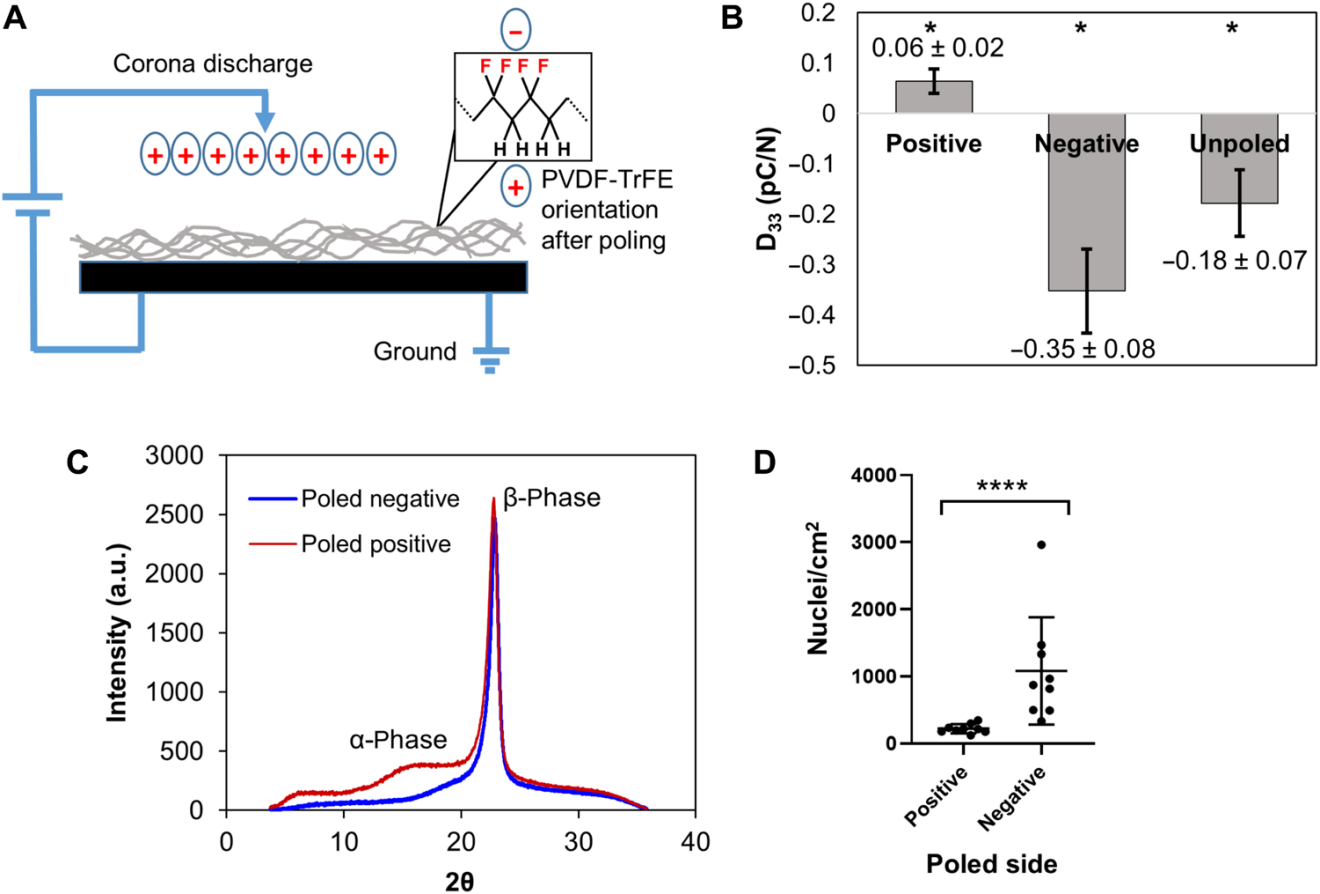
Effect of dipole orientation in piezoelectric scaffold on cell adhesion. (**A**) Corona poling setup where positive ions are produced in the atmosphere, which orients the dipoles in the PVDF-TrFE scaffold, resulting in one side being negatively poled and the other side being positively poled. (**B**) Piezoelectric coefficient (D_33_) of unpoled, poled positive, and poled negative microfiber randomly oriented PVDF-TrFE scaffolds. The sign of the coefficient indicates the direction of polarity. Values are means ± SD (*n* = 5). All groups are significantly different (*P < 0.05). (**C**) X-ray diffraction characterization of the effect of corona poling on the β-phase structure of the positive and negative surfaces of poled PVDF-TrFE scaffolds. a.u., arbitrary units. (**D**) Quantification of cell nuclei found on poled positive and poled negative surfaces. Center line and error bars indicate means ± SD from nine independent biological replicates for each type of surface. ****P < 0.0001 as determined by the Mann-Whitney test.

A distinctive feature of the PVDF-TrFE scaffolds is that they are piezoelectric (*41*), similar to cellulose found in the cell wall (*42*). Cellulose’s piezoelectric behavior, initially found in wood (*43*), is due to the dipole orientation in the molecular chains (*44*). Corona poling was used to enhance the piezoelectric properties of PVDF-TrFE scaffolds (Fig. 6A). Corona poling is a process where the scaffold is exposed to a high electric field, which then orients the dipoles more uniformly in PVDF-TrFE (*45*). Poling of PVDF-TrFE results in a significant change in the piezoelectric coefficient, D_33_, as compared to the unpoled PVDF-TrFE scaffold (Fig. 6B), where the negative and positive signs of the D_33_ value indicate the direction of polarity. X-ray diffraction analysis showed that while the positively poled PVDF-TrFE (positive D_33_ value) has both α-phase (nonpiezoelectric) and β-phase (piezoelectric) crystalline structures, the negatively poled PVDF-TrFE (negative D_33_ value) has essentially only β-phase (Fig. 6C), consistent with its enhanced piezoelectric activity. BY-2 cells cultured with poled PVDF-TrFE scaffolds showed that cells strongly prefer to adhere to the negatively poled than the positively poled side of the scaffold (Fig. 6D), indicating that the direction of the dipole can influence adhesion. In addition, the magnitude of the D_33_ value is also significantly higher on the negatively poled side as compared to the positively poled side. Notably, other properties such as fiber diameter, spacing, Young’s modulus, and relative hydrophobicity were not affected by poling in comparison to unpoled scaffolds (fig. S2).

### Cell wall pectins mediate the adhesion of plant cells to scaffolds

**Fig. 7.**
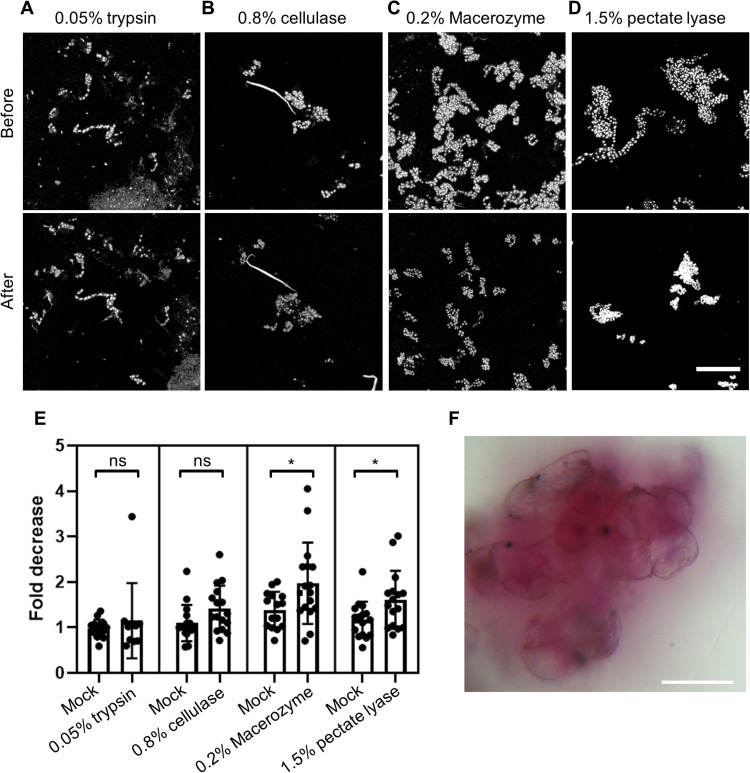
Pectin contributes to attachment of cells to scaffolds. (**A** to **D**) Maximum intensity projections of DAPI-stained BY-2 nuclei before and after 1-hour, room temperature treatment with 0.05% trypsin (A), 0.8% cellulase (B), 0.2% Macerozyme (C), and 1.5% pectate lyase (D). Scale bar, 250 μm. (**E**) Plot of fold decrease in the abundance of cell nuclei on scaffolds treated with 0.05% trypsin, 0.8% cellulase, 0.2% Macerozyme, and 1.5% pectate lyase treatments compared to mock treatments (trypsin, *n* = 14; cellulase, Macerozyme, and pectate lyase, *n* = 16). *P < 0.05 as determined by the Mann-Whitney test. (**F**) Pectin staining with ruthenium red of BY-2 cells on a microfiber randomly oriented PVDF-TrFE scaffold. Scale bar, 50 μm.

In animals, cell-cell and cell-matrix interactions are mediated by transmembrane adhesion proteins such as integrins. However, plant cells are surrounded by a micrometer-scale thick cell wall that prevents transmembrane proteins from being able to directly mediate cell-cell and cell-matrix interactions. Instead, plant cells physically interact with their environment through the cell wall. To determine which cell wall component underlies the adhesion of BY-2 cells to scaffolds, we treated cells attached to unpoled NRP scaffolds with enzymes that degrade specific components of the cell wall. We measured the abundance of cell nuclei before and after 1 hour of enzyme treatment to assess whether the treatment detached cells compared to mock-treated scaffolds. We intentionally did not use longer enzyme treatments to avoid confounding effects due to gross disruption of cell wall structure by prolonged digestion. Treatment with either 0.05% trypsin or 0.8% cellulase did not significantly decrease the cell nuclei abundance on scaffolds compared to mock treatment (Fig. 7, A, B, and E). However, treatment with 0.2% Macerozyme resulted in an approximately twofold decrease in the cell nuclei abundance on scaffolds compared to mock treatment (Fig. 7, C and E). Because Macerozyme is a mixture of pectinase and small amounts of cellulase and hemicellulase, we treated cells with pectate lyase to determine whether pectin degradation alone is sufficient to release cells. We saw that treatment with 1.5% pectate lyase also resulted in an approximately twofold decrease in the cell nuclei abundance on scaffolds compared to mock treatment (Fig. 7, D and E). Furthermore, staining of BY-2 cells on PVDF-TrFE scaffolds with a pectin-labeling dye, ruthenium red, showed diffuse red staining surrounding BY-2 cells (Fig. 7F), indicating that pectin on the surface of BY-2 cells might facilitate their attachment to scaffolds. Consistent with this observation, we found that BY-2 cells are in close contact with scaffold fibers (fig. S1, A and B). In some cases, we also observed apparent attachment sites between individual BY-2 cells and scaffold fibers (fig. S1C).

## DISCUSSION

The formation of organoids in microfluidic devices was made possible in mammalian systems by the development of natural and synthetic matrices that mimic the endogenous cell environment. These tools greatly accelerated discovery of fundamental mechanisms for the formation and function of specific three-dimensional tissue constructs and their application for tissue regeneration and repair. Plant-derived materials have proved to be useful for animal tissue engineering. For example, various natural and derivatized plant cell wall components have been used as biocompatible fibrous matrices for the adhesion and proliferation of mammalian cells (*46*–*48*). In addition, layers of paper containing hydrogels seeded with cells have been used to create three-dimensional mammalian tissue structures (*49*). Strikingly, decellularized whole leaves have been used as vascularized scaffolds for mammalian tissue engineering (*50*).

Here, we sought to apply animal tissue engineering methods to plant sciences. Currently, in vitro analyses of plant morphogenetic and physiological processes are conducted primarily using cultured plant cells (*35*). Recently, isolated plant cell protoplasts cultured in microwells were used to study subcellular events underlying plant cell morphogenesis (*32*, *51*). However, these approaches are not amenable to recapitulating multicellular plant tissues and development in vitro. Here, we describe the first scaffolds that support the adhesion of intact plant cells without causing extensive toxicity or perturbing cell morphology, division, and growth. This advance is a critical step toward culturing plant tissues in vitro to study how interactions between plants cells and their environment affect cell shape, differentiation, and tissue patterning.

PVDF-TrFE scaffolds mimicked the fibrous structure of plant cell walls, which can vary in dimension and orientation (*33*, *34*). We found that cell attachment was consistently higher on scaffolds with nanosized fibers than scaffolds with micrometer-sized fibers, with the highest number of cells attached to scaffolds with aligned nanosized fibers. Fiber dimension is known to affect the adhesion of animal cells. For example, adhesion and proliferation of mesenchymal cells is better on nanosized fibers compared to micrometer-sized fibers (*52*, *53*). Changes in the expression of integrins and focal adhesion kinases are thought to underlie the preference of animal cells for nanosized fibers (*54*, *55*). Why plant cells prefer to attach to aligned nanosized PVDF-TrFE fibers remains unknown. Cellulose microfibrils, the most prominent fibrous element of plant cell walls, are nanosized and are typically coaligned within a given strata of the cell wall (*33*, *34*). Therefore, aligned nanosized PVDF-TrFE fibers more closely mimic the physical features of the plant cell wall, which might somehow promote cell adhesion.

All scaffolds used in this study were relatively hydrophobic. Previous work has demonstrated that PVDF-TrFE scaffolds consist of the β-crystalline phase, which is the polar piezoelectric phase, but also other nonpolar crystalline and amorphous phases exist (*41*), which can contribute to its overall relatively hydrophobic property. Attachment of plant cells to hydrophobic scaffolds in our work is similar to the interaction of plant cells to hydrophobic PET microfiber infused with PLA nanofiber scaffolds (*38*). While the physiological relevance of scaffold hydrophobicity is unclear, it is possible that this property mimics hydrophobic surfaces of cellulose microfibrils and other cell wall materials (*56*).

The piezoelectric property of the scaffolds may also influence plant cell attachment. Upon corona poling of the scaffolds, cells almost exclusively attached to the negatively poled scaffold having the higher piezoelectric coefficient. Piezoelectric activity is a fundamental property of cellulose (*57*) and wood (*58*), but the implications of this property to plant cells are largely unknown. In our experiments, cells were cultured with scaffolds in liquid medium on a rotary shaker. It is possible that fiber movement in the fluid flow of the surrounding medium results in an electrical potential or streaming potential, as recorded in other culture environments (*41*), that acts as a signal for plant cell adhesion. Conversely, plant cells might be responsive to an electric charge generated in scaffolds by cell adhesion. In any case, it will be interesting to study whether the piezoelectric activity of cell walls contributes to strengthening of cell-cell adhesion and electrical signaling between cells in growing plant tissues.

The PVDF-TrFE scaffolds closely emulate fundamental cell wall properties such as a fibrous structure, hydrophobicity, and piezoelectricity. Therefore, successful scaffolds for plant cells cannot simply be passive structural materials, but rather, they need to mimic the distinctive chemical and physical characteristics of plant cell walls. Notably, BY-2 cells adhered and grew on these scaffolds as liquid suspension cultures and as callus cells, indicating that these scaffolds are suitable for both liquid and solid culture of plant cells. While adhesion to the scaffold did not significantly affect the overall cell morphology and cortical microtubule organization, cell size and certain microtubule dynamic behaviors were different in attached cells compared to control cells grown in the absence of a scaffold. These differences might be due to exhaustion of nutrients and accumulation of toxic metabolites in the 1-ml volumes used to grow cells on scaffolds. Alternatively, attachment to a scaffold might reduce the cell growth rate and alter microtubule dynamics. Development of a continuous culture system with these scaffolds should help to distinguish between these possibilities.

While animal cells adhere to the extracellular matrix through transmembrane proteins like integrins, plant cells adhere to each other through the pectin-rich middle lamella. Through enzymatic treatments, we determined that pectin, but not proteins or cellulose, plays a major role in adhering plant cells to scaffolds, although we cannot exclude the possibility that other cell wall components such as hemicelluloses might also contribute. Consistent with these data, ruthenium red staining demonstrated that BY-2 cells have abundant pectin on their surface. Our findings agree with previous work that indicated that BY-2 cells produce a layer of pectin after subculture (*59*). Additional work is required to uncover the mechanism by which pectin mediates the adhesion of plant cells to scaffolds. One possibility is that calcium and/or borate ions might cross-link pectin to scaffold fibers similar to the way these ions mediate pectin cross-linking in the middle lamella (*28*, *60*). A pectin-based adhesion mechanism is promising for transferring this technology to other plant cells, since pectin is found in all land plants. Furthermore, since the adhesion of BY-2 cells to scaffolds is reminiscent of the way cells adhere to each other in plant tissues, our scaffolds should be suitable for plant organoid development.

As fabricated in this study, our scaffolds are best suited to replicate epidermal tissue with cells adhered to the surface of a scaffold. However, by controlling the three-dimensional shape of the scaffold, it should be possible to mimic internal plant tissues and complex tissue geometries in vitro. In addition, this study provides a strong foundation to rationally explore other polymer types and modifications to either improve or tune plant cell attachment in the future.

## METHODS

### Fabrication of PVDF-TrFE scaffolds

Scaffolds were prepared using the electrospinning technique. Random or aligned fibrous PVDF-TrFE scaffolds were fabricated by modifying previous methods as described (*61*). Briefly, 17.5 or 20% (w/v) solutions of PVDF-TrFE (70/30) (400 kDa, polydispersity index (PDI) of 2.1, Solvay Solexis Inc., Thorofare, NJ) in methyl ethyl ketone (Sigma-Aldrich Inc.) were loaded into a syringe with an 18-gauge needle. To generate nanosized fibers, a voltage of 28 kV was applied to the tip of the needle when electrospinning 17.5% (w/v) PVDF-TrFE solutions. Random fibers were connected on a grounded plate, and aligned fibers were collected on a grounded drum at 3200 rpm. The solution was expelled from the syringe at a flow rate of 3 ml/hour at a distance of 40 cm from the collector. Similarly, to generate micrometer-sized fibers, a voltage of 22.5 kV was applied to the tip of the needle when electrospinning 20% (w/v) PVDF-TrFE solutions. Random fibers were connected on a grounded plate, and aligned fibers were collected on a grounded rotating drum at 2600 rpm. The solution was expelled from the syringe at a flow rate of 3.3 ml/hour, 30 cm from the collector. After electrospinning, the scaffolds were annealed as described in previous methods (*41*).

### Mechanical testing

Tensile testing was performed to determine the Young’s modulus and maximum tensile stress of PVDF-TrFE matrices. Tensile tests were performed using a mechanical tester (Instron, MA, USA), as previously described (*41*). Briefly, matrices were cut into 70 mm–by–10 mm strips. A gauge length of 40 mm was used, and the strips were subjected to a displacement rate of 10 mm/min. All scaffolds were sterilized with 100% ethanol for 20 min and immersed in phosphate-buffered saline for 5 min before testing. A sample size of 10 per group was used unless otherwise noted.

### Goniometer measurements

Scaffolds were cut into 1 cm–by–1 cm samples (*n* = 4 per group). Wettability measurements were performed by air-water contact angle using a goniometer built in-house. The scaffolds were mounted onto a glass slide. Water (20 μl) was dropped on the scaffold from a height of 1 cm, and the contact angle was recorded using a Sony HDR-XR260V camera.

### X-ray diffraction

X-ray diffraction scans were collected using a Bruker D8 Discover diffractometer equipped with a general area detection system and Co radiation generated at 40 kV and 35 mA and a collection time of 10 min.

### Corona poling of the PVDF-TrFE scaffolds

Corona poling was performed using a custom setup. Depending on the thickness of the matrix used, the PVDF-TrFE matrices were heated using a heating plate to a temperature of 47° to 55°C for 30 to 60 min. A metal grid connected to a primary voltage source (set to 2 kV) was suspended 2 cm above the heated matrix. A needle connected to a secondary voltage (set to 14 kV) was placed above the grid with the needle tip oriented in the direction of the grid. Charge was discharged for 3 to 6 hours.

### Measuring piezoelectric coefficient (D_33_) of PVDF-TrFE scaffolds

Scaffolds were cut into 1 cm–by–1 cm samples from each group (*n* = 5 per group). D_33_ values were measured using a piezometer (Piezotest, D_33_ Piezometer System, Singapore). Each matrix was placed onto a piezometer, and the D_33_ value was measured at a dynamic force of 0.25 N at 110 Hz.

### SEM of scaffolds

Scaffolds were sputter-coated with gold-palladium (EMS 150 TES, PA, USA) and viewed using an SEM (JSM-7900F JEOL, USA) with an accelerating voltage of 2 to 3 kV. Two images in three different areas of each scaffold were captured. Fiber diameters and inter-fiber spacing were measured at *n* = 15 per image using ImageJ software, as previously described (*48*). The percent alignment for aligned fiber matrices was measured as previously described (*48*). Briefly, a line was drawn perpendicular to a fiber, chosen at random, in the SEM image. Ten angles were measured between the line and the fibers. The absolute deviation value (ADV) of the measured angles from 90° was calculated and averaged. The percent alignment was calculated as

$$\%\;\text{Alignment}=(\frac{90{}^{\circ}-\text{ADV}}{90{}^{\circ}})*100\%$$

### BY-2 cell culture and transformation

Tobacco BY-2 cell cultures were maintained in 100 ml of liquid medium [Murashige and Skoog basal salts (4.3 g/liter), myo-inositol (100 mg/liter), thiamine (1 mg/liter), 2,4-D (0.2 mg/liter), KH_2_PO_4_ (255 mg/liter), and 3% sucrose, pH 5.0] in 500-ml VWR polycarbonate Erlenmeyer flasks at 25°C on a rotary platform shaker at 130 rpm. Cells were subcultured weekly using a ratio of 1:20 of cell culture to fresh liquid medium.

BY-2 cells were stably transformed using *Agrobacterium tumefaciens*–mediated transformation. Briefly, wild-type BY-2 cells were cocultivated with Agrobacteria carrying *ubiquitin-10* promoter–driven mEGFP-TUB6 in a pGAG vector for 2 days at 25°C. Stably transformed cells were selected using kanamycin (25 mg/liter) and verified for expression using confocal microscopy.

### Cell attachment and callus growth experiments on scaffolds

Scaffolds were cut into small pieces (approximately 0.75 cm by 1 cm) and sterilized by submerging in 100% ethanol for 1 hour with shaking to remove air bubbles from the scaffolds. Scaffolds were then dried in a laminar flow hood and rinsed thrice with sterile ultrapure water and once with fresh BY-2 culture medium. Sterile scaffolds were coincubated with 4-day-old BY-2 cell cultures in 1 ml of liquid medium in 1.8-ml screwcap cryotubes (Thermo Fisher Scientific, 368632), placed on a nutator for 4 days at 50 rpm, and then washed with 25 ml of fresh medium in a 50-ml centrifuge tube overnight on a nutator at 50 rpm to remove unbound cells.

For callus growth experiments, scaffolds were sterilized and rinsed using the above method and then placed on solid BY-2 medium [Murashige and Skoog basal salts (4.3 g/liter), myo-inositol (100 mg/liter), thiamine (1 mg/liter), 2,4-D (0.2 mg/liter), KH_2_PO_4_ (255 mg/liter), 3% sucrose (pH 5.7), 0.8% phytagel, and carbenicillin (100 μg/ml)] in 100 mm–by–100 mm square petri dishes. Four-day-old BY-2 cells were centrifuged at 4000*g* for 10 min to concentrate, and 10 μl of cells was pipetted onto scaffolds or directly on the solid medium as control. Plates were wrapped with micropore tape and incubated at 25°C.

### Imaging and image analysis

Fluorescence microscopy was performed on a Nikon A1si confocal microscope. For cell counting, cells were stained with DAPI (1 μg/ml; AnaSpec Inc., AS-83210) in BY-2 medium for 10 min, briefly rinsed in fresh medium, and then imaged with a 403-nm diode-pumped solid-state laser (Melles Griot) and a 425- to 475-nm emission filter. For initial screening and cell viability staining, cells were stained with FM 4-64 (3 μg/ml; Tocris Bioscience, 5118) in BY-2 medium and then imaged with a 560-nm diode-pumped solid-state laser (Melles Griot) and a 575- to 620-nm emission filter.

For live/dead cell staining, cells were stained in fluorescein diacetate (2.5 μg/ml; Sigma-Aldrich, F7378) and propidium iodide (10 μg/ml; Adipogen Corp., CDX-P0023) for 15 min and imaged using a 488-nm argon-ion laser (Melles Griot) and a 560-nm diode-pumped solid-state laser (Melles Griot) and 500- to 550-nm and 575- to 620-nm emission filters. To monitor the effect of scaffold binding on cell viability, sterilized NRP scaffolds were coincubated with BY-2 cells 2 days after subculture (day 0). Individual scaffolds were stained 1, 2, and 3 days after coincubation and compared to the staining of unattached cells from the same flasks. BY-2 cells grown in the absence of a scaffold were used as a control. For cell shape analysis, cells were stained in calcofluor (1 mg/ml; Sigma-Aldrich, F3543) for 1 min and then imaged with a 403-nm diode-pumped solid-state laser (Melles Griot) and a 425- to 475-nm emission filter. For cell division and microtubule dynamics imaging, cells were imaged using a 488-nm argon-ion laser (Melles Griot) and a 500- to 550-nm emission filter. Imaging intervals were 60 s for cell division and 4 s for microtubule dynamics.

All images were processed using ImageJ. Nuclei counts were obtained using an ImageJ macro that took maximum intensity projections, converted them to 8-bit, and then used the contrast method of the Auto Local Threshold function (radius, 50) to create thresholded images of nuclei. The macro then ran Analyze Particles with a 20- to 200-pixel^2^ size filter to count the number of nuclei. To determine the effect of enzyme treatments on cell adhesion, all images were converted to maximum intensity projections and then combined into a single stack for uniform processing. The intensities of the images in the stack were then normalized using the Enhance Contrast function (0% saturation). The integrated signal intensities of the images were measured and used to calculate the fold decrease by dividing the before-treatment intensity by the after-treatment intensity. To measure cell size, the outline of cells was defined manually, and their area and perimeter were measured using ImageJ. The aspect ratio of cells was measured by dividing the length of each cell by its width. Microtubule array orientation and anisotropy were measured using the FibrilTool plugin (*40*). Kymograph analysis of cortical microtubule dynamics was performed as previously described (*62*). Kymographs were generated only with microtubules whose entire length stayed in focus during the observation period and showed at least two assembly dynamics phases.

### Enzyme treatments

Cell nuclei on nanofiber randomly oriented PVDF-TrFE scaffolds were imaged after staining for 10 min with DAPI (1 μg/ml), then placed in a 1.7-ml microcentrifuge tube with 1 ml of BY-2 medium containing either 0.05% trypsin (Sigma-Aldrich, T2601), 0.8% cellulase (Duchefa Biochemie, C8001), 0.2% Macerozyme R-10 (Duchefa Biochemie, M8002), or 1.5% pectate lyase (Megazyme, E-PLYCJ), and shaken at room temperature for 1 hour. Mock treatments used BY-2 medium alone. Scaffolds were washed in 25 ml of fresh medium three times and then stained for 10 min with DAPI for imaging.

### Environmental SEM

Scaffolds were coincubated with BY-2 cells and washed according to our standard protocol, dried on one side with a Kimwipe, adhered to a mount, and imaged using a Zeiss Evo10 environmental SEM. Vacuum-chamber vapor point varied between 80 and 100 Pa, and electron high tension value varied between 16 and 20 kV.

### Statistical analysis

Statistical analyses were performed using GraphPad Prism 8 (GraphPad Software, San Diego, CA, USA). Normality was determined using Anderson-Darling, D’Agostino and Pearson, Shapiro-Wilk, and Kolmogorov-Smirnov tests. For nonnormal datasets, the Kruskal-Wallis test with a Dunn’s test was used to determine statistical significance (*P* < 0.05) for multiple comparisons, and the Mann-Whitney test with Bonferroni correction was used for comparison between two groups. For quantitative materials characterization data, statistical analyses were performed in SPSS Statistics version 25 (IBM, Armonk, NY, USA). One-way analysis of variance (ANOVA) was used to determine statistical significance (*P* < 0.05). Normality was determined using the Shapiro-Wilk test and Levene’s equal variance test. Tukey’s post hoc test was used for statistical differences at *P* < 0.05. Values are reported as means ± SD, unless otherwise mentioned.
