# Supplementary material for: Plant cell adhesion and growth on artificial fibrous scaffolds as an in vitro model for plant development
Source: Sci Adv. 2021 Oct 20;7(43):eabj1469. doi: 10.1126/sciadv.abj1469 (PMC8528414; doi:10.1126/sciadv.abj1469)
Supplement: Supplementary file 1 — Figs. S1 and S2 [file sciadv.abj1469_sm.pdf]

Science Advances
AAAS

Supplementary Materials for

Plant cell adhesion and growth on artificial fibrous scaffolds as an in vitro model for plant development

Ryan Calcutt, Richard Vincent, Derrick Dean*, Treena Livingston Arinzeh*, Ram Dixit*

*Corresponding author. Email: arinzeh@njit.edu (T.L.A.); ddean@alasu.edu (D.D.); ramdixit@wustl.edu (R.D.)

Published 20 October 2021, *Sci. Adv.* **7**, eabj1469 (2021)
DOI: 10.1126/sciadv.abj1469

This PDF file includes:

Figs. S1 and S2

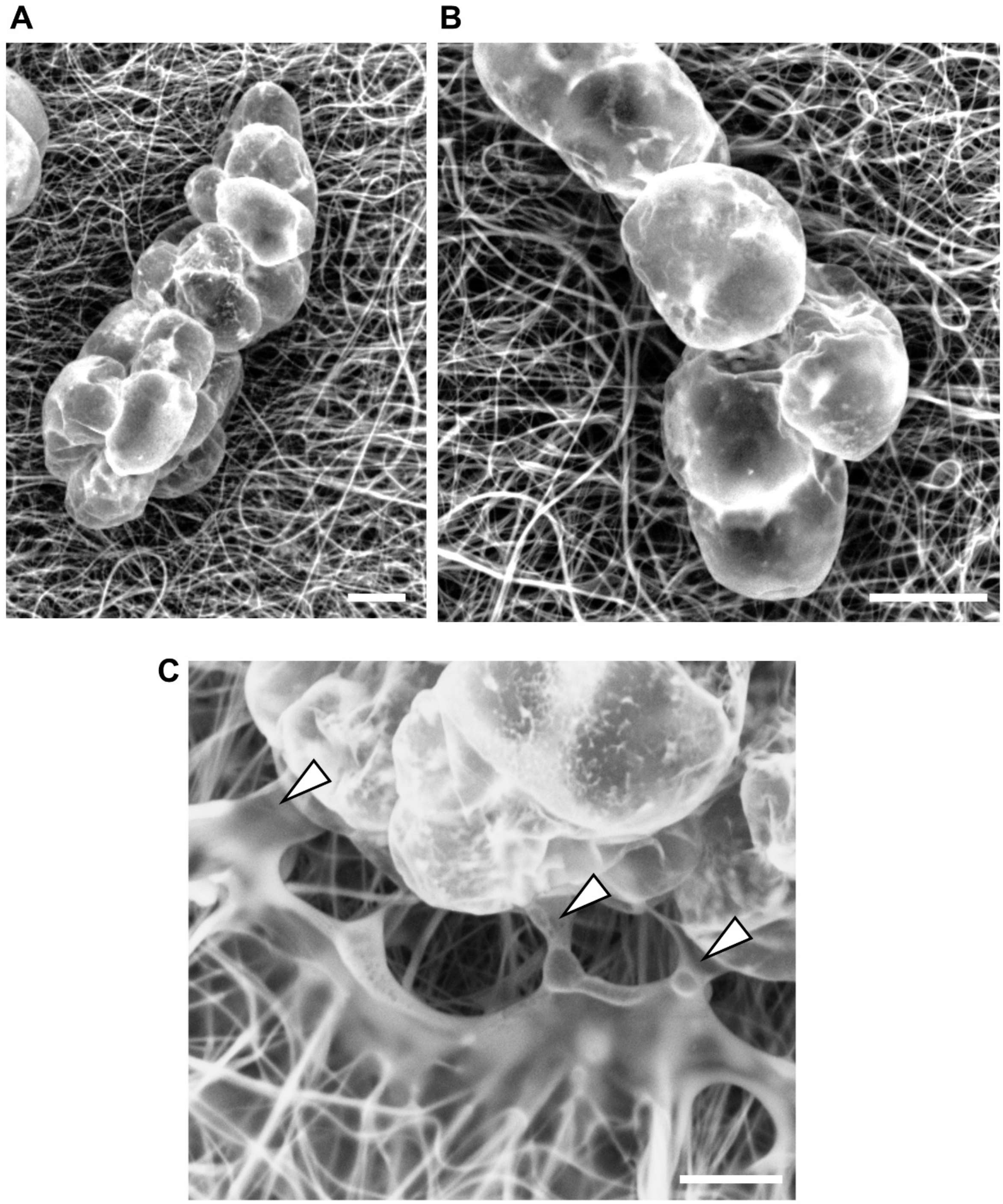

Fig. S1. High magnification images of BY-2 cells on NRP scaffold. (**A, B**) Environmental scanning electron micrographs of BY-2 cells on nanofiber randomly oriented PVDF-TrFE scaffolds. Scale bar = 20 µm. (**C**) Image of apparent attachment sites (arrowheads) between BY-2 cells and scaffold fibers. Scale bar = 10 µm

A

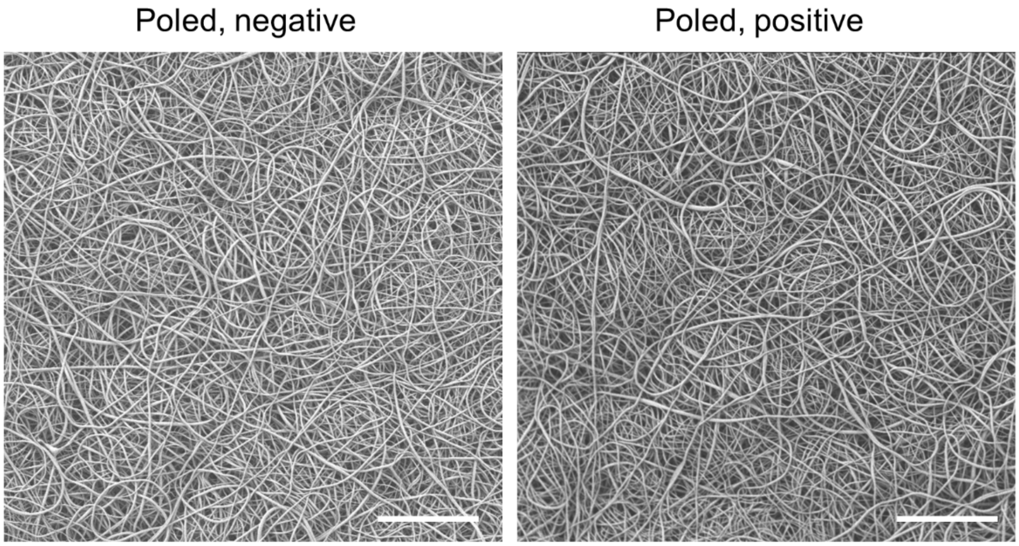

B

	Poled negative	Poled positive
**Average fiber diameter (µm)**	2.64 ± 0.51	2.65 ± 0.54
**Inter-fiber spacing (µm)**	30.35 ± 9.17	37.21 ± 12.43
**Percent fiber alignment (%)**	N/A	N/A
**Young's modulus (MPa)**	7.11 ± 1.03	7.11 ± 1.03
**Contact angle (°)**	132.4 ± 4.4	136.7 ± 5.6

Fig. S2. Physical properties of poled PVDF-TrFE scaffolds. (**A**) Scanning electron micrographs of poled negative and poled positive PVDF-TrFE surfaces. Scale bar = 100 µm. (**B**) Table showing the fiber diameter, inter-fiber spacing, fiber alignment, Young's modulus and air-water contact angle of the poled negative and poled positive PVDF-TrFE surfaces. Values are mean ± S.D. (n = 15 per group for the fiber measurements; n = 5 per group for the Young's modulus; n = 4 per group for air-water contact angle).
